# A Multicenter Study on Early Outcomes of Small-Incision Lenticule Extraction for Myopia

**DOI:** 10.1038/s41598-019-40805-1

**Published:** 2019-03-11

**Authors:** Kazutaka Kamiya, Masahide Takahashi, Tomoaki Nakamura, Takashi Kojima, Ikuko Toda, Maro Kariya

**Affiliations:** 1 School of Allied Health Sciences, Kitasato University, Kanagawa, Japan; 2 Department of Ophthalmology, University of Kitasato School of Medicine, Kanagawa, Japan; 3 Nagoya Eye Clinic, Department of Ophthalmology, Aichi, Japan; 4 Keio University, Tokyo, Japan; 5 Minami Aoyama Eye Clinic, Tokyo, Japan; 6 Shinjuku Kinshi Clinic, Tokyo, Japan

## Abstract

This study was aimed to investigate the early clinical outcomes of small-incision lenticule extraction (SMILE) to correct both myopia and myopic astigmatism at major clinical centers in Japan. This case series consisted of two hundred fifty-two eyes of 130 consecutive patients who underwent SMILE surgery (29.5 ± 6.3 years, mean age ± standard deviation), with spherical equivalents of −4.33 ± 1.61 D. We determined the safety, efficacy, predictability, stability, and adverse events of this procedure. Corrected distance visual acuity significantly improved, from −0.18 ± 0.04 preoperatively to −0.19 ± 0.07 logMAR postoperatively (paired t-test, p < 0.001). Uncorrected distance visual acuity also significantly improved, from 1.05 ± 0.26 preoperatively to −0.15 ± 0.11 logMAR postoperatively (p < 0.001). 88% and 98% of eyes were within ± 0.5 and 1.0 D of the targeted correction, respectively. Changes in manifest spherical equivalent from 1 week postoperatively were 0.02 ± 0.35 D (p = 0.127). No vision-threatening complications were observed in any of the cases. SMILE performed well in the correction of myopic refractive errors, and we experienced no severe complications in this series, indicating its feasibility as a surgical option for the treatment of these eyes.

## Introduction

The femtosecond laser can precisely cut corneal tissues with less thermal damage, and thus has not only been applied for flap making for laser *in situ* keratomileusis (LASIK)^1^, but also for all-in-one refractive surgery called small incision lenticule extraction (SMILE) without flap creation^2,3^. SMILE has been reported to be longitudinally good in terms of safety, efficacy, predictability, and stability, to correct both myopia and myopic astigmatism. However, most were single center studies with a small sample size. As far as we can ascertain, there have been no other published multicenter studies on the outcomes of SMILE in a large cohort of patients. It may give intrinsic insights on the whole image and the further refinement of SMILE, since it is less influenced by the individual surgeon’s skills and experiences at a single medical center. The objective of this study is to retrospectively assess the early outcomes of SMILE for myopic refractive errors, in a cohort of patients presenting at major institutions in Japan.

## Results

### Patient Population

Preoperative patient demographics are listed in Table 1. The number of eyes at follow-up examination was 252 eyes (100%) at 1 day, 250 eyes (99%) at 1 week, 243 eyes (96%) at 1 month, and 252 eyes (100%) at 3 months, postoperatively.

**Table 1:** Preoperative demographics of the study population in eyes undergoing small incision lenticule extraction (SMILE).

Demographic Data	
Age (years)	29.5 ± 6.3 years (95% CI, 17.2 to 41.8 years)
Gender	Male: Female = 148: 104
LogMAR UDVA	1.05 ± 0.26 (95% CI, 0.53 to 1.57)
LogMAR CDVA	−0.18 ± 0.04 (95% CI, −0.25 to −0.09)
Manifest spherical equivalent (D)	−4.33 ± 1.61 D (95% CI, −1.17 to −7.49 D)
Manifest cylinder (D)	0.64 ± 0.51 D (95% CI 0.36 to 0.64 D)
Mean keratometric reading (D)	43.1 ± 1.5 D (95% CI, 40.2 to 46.0 D)
Central corneal thickness (μm)	545.8 ± 29.2 μm (95% CI, 488.6 to 603.0 μm)

CI = confidence interval; logMAR = logarithm of the minimal angle of resolution; UDVA = uncorrected distance visual acuity; CDVA = corrected distance visual acuity; D = diopter.

### Safety and Efficacy

**Figure 1:**
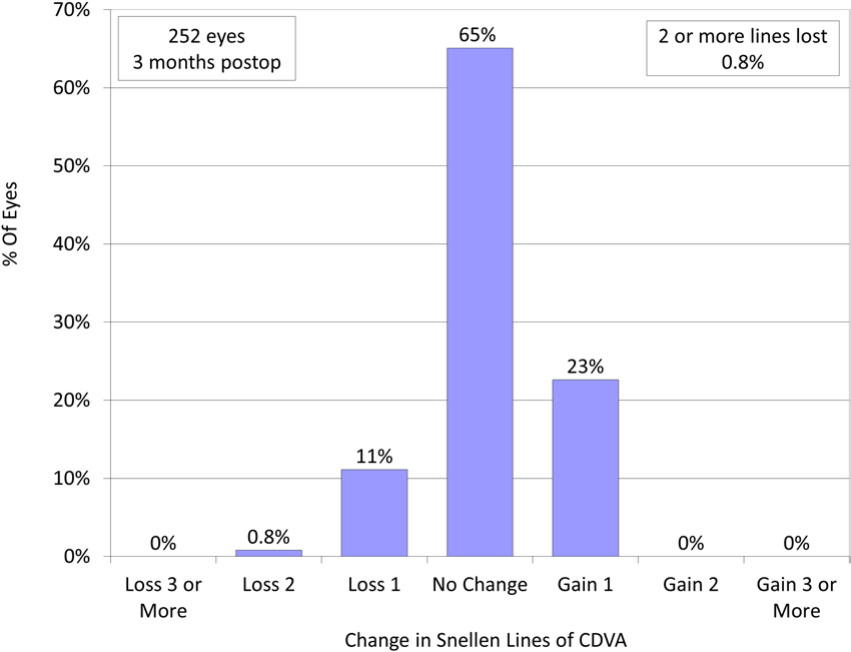
Changes in corrected distance visual acuity (CDVA) after small incision lenticule extraction (SMILE).

**Figure 2:**
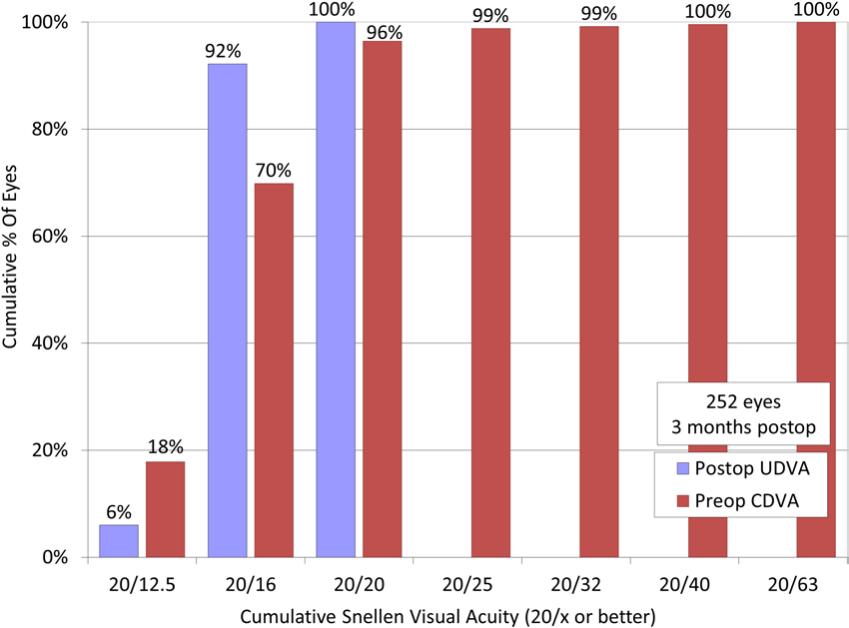
Cumulative percentages of eyes attaining specified cumulative levels of uncorrected distance visual acuity (UDVA) after small incision lenticule extraction (SMILE).

Logarithm of the minimal angle of resolution (LogMAR) of corrected distance visual acuity (CDVA) improved significantly from −0.18 ± 0.04 preoperatively to −0.19 ± 0.07 postoperatively (paired t-test, p < 0.001). At 3 months postoperatively, 164 eyes (65%) showed no change in CDVA, 57 eyes (23%) gained 1 line, 28 eyes (11%) lost 1 line, and 2 eyes (0.8%) lost 2 lines (Fig. 1). LogMAR of uncorrected distance visual acuity (UDVA) also improved significantly from 1.05 ± 0.26 preoperatively to −0.15 ± 0.11 postoperatively (p < 0.001). 92% and 100% of eyes had UDVA of 20/16 or better and 20/20 or better, respectively (Fig. 2).

### Predictability and Stability

**Figure 3:**
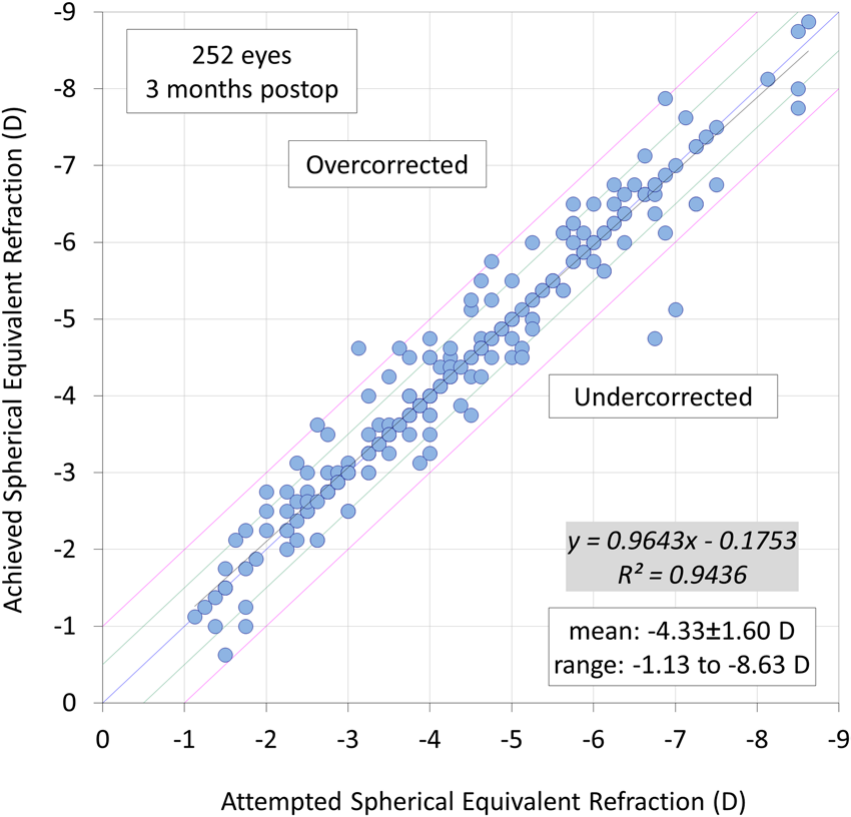
A scatter plot of the attempted versus the achieved manifest spherical equivalent correction after small incision lenticule extraction (SMILE).

**Figure 4:**
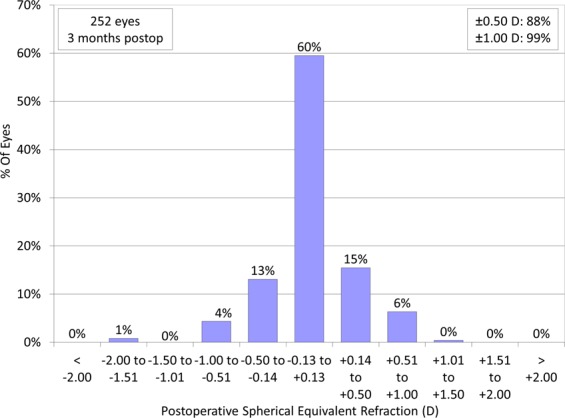
Percentages of eyes within different diopter ranges of the attempted correction (spherical equivalent) after small incision lenticule extraction (SMILE).

**Figure 5:**
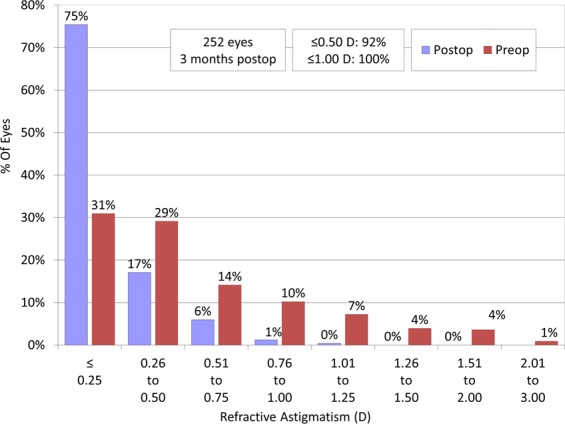
Percentages of eyes within different diopter ranges of refractive astigmatism before and 3 months after small incision lenticule extraction (SMILE).

**Figure 6:**
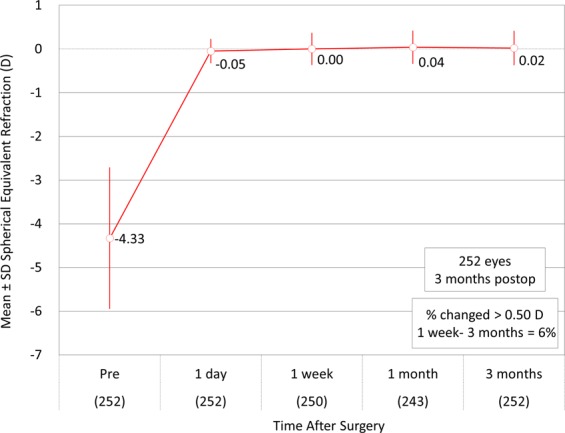
Time course of manifest spherical equivalent after small incision lenticule extraction (SMILE).

Figures 3–5 show a scatter plot of the attempted versus the achieved equivalent correction, the refractive accuracy, and the refractive astigmatism. At 3 months postoperatively, 88% and 98% were within ± 0.5 and 1.0 diopters (D) of the attempted correction, respectively. Figure 6 shows the changes over time in the subjective refraction. Changes in refraction from 1 week to 3 months postoperatively were 0.02 ± 0.35 D. We found no significant change in refraction between 1 week and 3 months postoperatively (p = 0.127).

### Complications

Suction loss occurred in 3 eyes (1.2%), but the surgeries were successfully completed after reattaching the contact glass. Subconjunctival hemorrhage occurred in 7 eyes (2.8%). Transient interface haze and diffuse lamellar keratitis (grade 1) developed in 23 eyes (9.1%) and 2 eyes (0.8%), respectively, during the first postoperative month. All these eyes were monitored without additional surgical intervention, and gradually resolved after that. Circle enhancement and laser-assisted subepithelial keratectomy, due to undercorrection, were required in 3 eyes (1.2%) and 1 eye (0.4%), respectively. All of these eyes had a UDVA of 20/20 or more postoperatively. No keratectasia, epithelial ingrowth, or any other severe complications were observed.

## Discussion

This study shows that SMILE performed well in all measures of safety, efficacy, and predictability for the correction of myopia and myopic astigmatism, and that no severe complications occurred in any of the cases. To the best of our knowledge, this is the first multicenter study to investigate the visual and refractive outcomes and the adverse events of SMILE in a large number of patients presenting at major clinical centers in Japan. Our outcomes were assumed to be less dependent on the individual surgical skills and experiences of refractive surgeons, and thus were clinically helpful for understanding the whole image of SMILE in Japan.

Previous major studies in a large number of patients undergoing SMILE are summarized in Table 2^4–13^. Our findings were comparable with, or slightly better than, those in previous studies in terms of safety and efficacy, possibly because the amount of myopic correction is relatively low in this series. Our findings were also comparable with those in previous studies in terms of predictability^4–10,12,13^, except for one long-term (5-year) study^11^. In terms of stability, we found no significant refractive regression from 1 week to 3 months after SMILE in this study. However, we cannot conclude on long-term refractive stability of SMILE at this time, since the follow-up period was up to 3 months in this case series. Blum *et al*.^11^ demonstrated that the mean long-term regression was 0.48 D. Han *et al*.^14^ showed that the manifest spherical equivalent was −0.09 ± 0.39 D 4 years postoperatively and did not significantly change from −0.01 ± 0.33 D 6 months postoperatively. We are awaiting a further long-term study to investigate whether or not myopic regression occurs in the late postoperative period.

**Table 2:** Previous studies on visual and refractive outcomes in a large cohort of patients undergoing small incision lenticule extraction (SMILE).

Author	Year	Eyes	Follow-up	Age	Spherical equivalent	Astigmatism	Safety	Efficacy	Predictability		Stability
			(months)	(years)	(D)	(D)	(logMAR CDVA)	(logMAR UDVA)	within ± 0.5D (%)	within ± 1.0D (%)	(D)
Sekundo *et al*.^4^	2011	91	6	35.6	−4.75 ± 1.56	0.78 ± 0.79	53% unchanged 35.6% gained ≥ 1 line 9.9% lost ≥ 1 line	83.5% ≤ 0.00 logMAR	80.2	95.6	−0.01 ± 0.49
Vestergaard *et al*.^5^	2012	279	3	38.1 ± 8.7	−7.18 ± 1.57	0.71 ± 0.50	−0.03 ± 0.07	73% ≤ 0.00 logMAR	77	95	−0.20 ± 0.39
Hijordal *et al*.^6^	2012	670	3	38.3 ± 8.3	−7.19 ± 1.30	0.60 ± 0.46	−0.049 ± 0.097	84% ≤ 0.10 logMAR	80.1	94.2	−0.25 ± 0.44
Ivarsen *et al*.^7^	2014	1574	3	38 ± 8	−7.25 ± 1.84	0.93 ± 0.90	−0.05 ± 0.10	—	—	—	−0.28 ± 0.52
Reinstein *et al*.^8^	2014	110	12	32.4 ± 5.7	−2.61 ± 0.54 (Low)	0.55 ± 0.38	66% unchanged 25% gained ≥ 1 line 9% lost 1 line	96% ≤ 0.00 logMAR	84	99	−0.05 ± 0.36
Pedersen *et al*.^9^	2015	87	36	37 ± 7.8	−7.30 ± 1.40	0.70 ± 0.60	−0.08 ± 0.11	0.03 ± 0.19	78%	90%	−0.39 ± 0.61
Hansen *et al*.^10^	2016	722	3	N.A.	−6.82 ± 1.66	0.83 ± 0.84	0.07 ± 0.03 gain	83% ≤ 0.10 logMAR	88%	98%	−0.37 ± 0.48
Blum *et al*.^11^	2016	56	60	42	—	—	−0.12	0.01	48.2%	78.6%	−0.375
Jin *et al*.^12^	2017	62	3	23.32 ± 4.54	−7.16 ± 0.93 (High)	0.91 ± 0.60	Index 1.06 ± 0.09	Index 0.98 ± 0.18	87%	95%	−0.20 ± 0.37
		103	3	24.34 ± 6.12	−4.34 ± 0.97 (Low, Moderate)	0.60 ± 0.51	Index 1.06 ± 0.09	Index 1.05 ± 0.10	100%	100%	0.01 ± 0.19
Torky *et al*.^13^	2017	94	6	29.09	−2.73 (Low)	0.25	Index 1.02 ± 0.16	Index 0.943 ± 0.18	89.3%	100%	0.00
		95	6	27.5	−4.25 (Moderate)	0.50	Index 1.05 ± 0.16	Index 0.976 ± 0.18	89.4%	97.8%	−0.10
		85	6	26.7	−7.25 (High)	0.50	Index 1.09 ± 0.16	Index 0.917 ± 0.18	88.2%	95.2%	−0.49
Current		252	3	29.5 ± 6.3	−4.33 ± 1.61	0.64 ± 0.51	−0.19 ± 0.07	−0.15 ± 0.11	88	98	0.02 ± 0.35

D = diopter; logMAR = logarithm of the minimal angle of resolution; CDVA = corrected distance visual acuity; UDVA = uncorrected distance visual acuity.

In this multicenter study, we included multiple refractive surgeons to perform SMILE. Most surgeons were experienced, but some were novice surgeons. Ivarsen *et al*.^7^ described that SMILE is a technically more demanding procedure than other keratorefractive techniques, and that the frequency of these complications appeared to depend on surgical skills and laser settings. Titiyal *et al*.^15^ stated that the flap-to-cap transition may be surgically challenging during the SMILE learning curve, even for experienced surgeons in performing LASIK surgery, and that most complications that result in delayed visual recovery are found during the first 50 cases. We thought it would be appropriate to include multiple refractive surgeons, in order to make this study less dependent on the individual skills and experiences of refractive surgeons, and to grasp the whole image of SMILE performed in Japan.

In another multicenter studies, we recently showed that posterior chamber phakic intraocular lens implantation offered good safety and efficacy outcomes, and yielded predictable and stable results, with no vision-threatening complications^16–18^. Although the degree of myopic correction is different among these studies, both SMILE and phakic intraocular lens implantation may be viable surgical options for the treatment of myopic refractive errors.

This study is burdened with two limitations. First, due to staffing limitations at each institution, the maximum follow-up was 3 months. More prolonged follow-up will be necessary to evaluate late-onset complications, such as long-term regression, or late-onset iatrogenic ectasia. Secondly, some patients dropped out during the 3-month observation period. Although only 1 to 4% of patients were lost, patient selection bias might be included in this study population.

In conclusion, our multicenter demonstrated that SMILE offered good safety and efficacy outcomes and yielded predictable results without the occurrence of severe complications, indicating that SMILE is one of the feasible surgical options to correct myopic refractive errors. This study was less dependent on the individual skills and experiences of single refractive surgeons, since it was performed in a multicenter setting. We assume that although this information is simple, it may be clinically helpful for grasping the whole image of SMILE.

## Methods

### Study Population

The study protocol was enrolled with the University Hospital Medical Information Network Clinical Trial Registry (000031473). Two hundred and fifty-two eyes of 130 consecutive patients (148 men and 104 women, mean age ± standard deviation (SD), 29.5 ± 6.3 years) who underwent SMILE for myopia and myopic astigmatism at 4 major institutions (Nagoya Eye Clinic, Minami Aoyama Eye Clinic, Shinjuku Kinshi Clinic, and Kitasato University Hospital) from January 2017 to December 2017, and who finished a 3-month observation period, were retrospectively reviewed from the clinical charts at each institute. This study was performed as a collaborative work of the Japan SMILE Study Group. The sample size in this study offered 97.7% statistical power at the 5% level in order to detect a 0.10-difference in logMAR of visual acuity, when the SD of the mean difference was 0.40.

The general inclusion criteria for this surgical procedure at each institution were unsatisfaction with spectacle or contact lens correction, manifest spherical equivalent of −1 to −9 D, manifest cylinder of 0 to 4 D, estimated total postoperative corneal thickness >400 μm, endothelial cell density ≥1800 cells/mm^2^, and no history of ocular surgery, progressive corneal degeneration, severe dry eye, cataract, glaucoma, or uveitis. We excluded eyes with keratoconus from the study by using the keratoconus screening test. In all eyes, the preoperative manifest refraction was selected as the target correction. Preoperatively, 1 day, and 1 week and 1, and 3 months postoperatively, we determined UDVA, CDVA, manifest spherical equivalent refraction, and manifest astigmatism. The study was approved by the Institutional Review Board of Kitasato University and followed the tenets of the Declaration of Helsinki. The requirement for informed consent for this retrospective study was waived by our Institutional Review Board.

### Surgical Procedure

**Figure 7:**
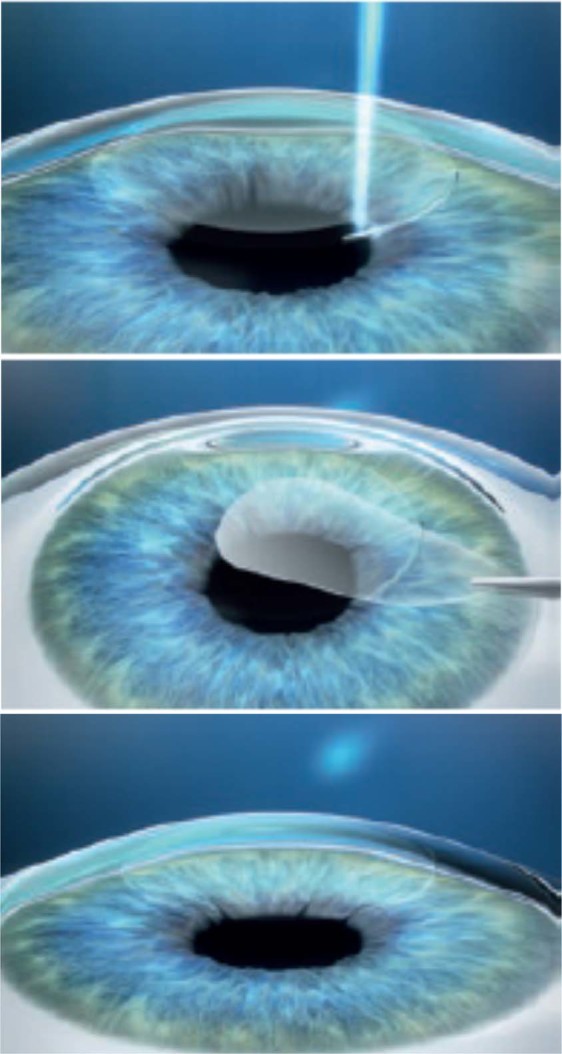
A schematic drawing of the small incision lenticule extraction (SMILE) procedure.

The standard SMILE procedure was conducted by multiple surgeons at each institution, using the VisuMax femtosecond laser system (Carl Zeiss Meditec, Jena, Germany) with a 500 kHz repetition rate, as described previously^19–21^. The following laser parameters were used: 120 μm cap thickness, 7.5 mm cap diameter, 6.5 mm lenticule diameter, 140 to 170 nJ power for lenticule making, a 2- to 3-mm side cut with angles of 90°. After laser treatment, a blunt spatula was inserted through the side cut over the top of the lenticule dissecting this plane followed by the bottom of the lenticule. After that, the lenticule was grasped and removed with forceps (Fig. 7). Postoperatively, steroidal and antibiotic medications were topically used 4 times daily for 2 weeks, and thereafter the frequency was steadily reduced.

### Statistical Analysis

All statistical analyses were conducted using a commercially available statistical software (BellCurve for Excel, Social Survey Research Information Co, Ltd., Tokyo, Japan). The paired t-test was used for statistical analysis to compare the pre- and post-surgical data, because the normal distribution of the data was confirmed by the Kolmogorov-Smirnov test. The results are described as mean ± SD, and a value of p < 0.05 was considered statistically significant.

## Data Availability

The datasets generated during and/or analyzed during the current study are available from the corresponding author on reasonable request.
